# Small extracellular vesicles from deferoxamine-primed stem cells promote angiogenesis and tissue integration

**DOI:** 10.20517/evcna.2026.48

**Published:** 2026-09-08

**Authors:** Jingyi Sang, Yuqing Dong, Xiaoyue Zhang, Xiaojing Yuan, Yike Gao, Rui Song, Xiya Liu, Linxue Zhang, Zuoying Yuan, Yuming Zhao

**Affiliations:** ^1^Department of Pediatric Dentistry, Peking University School and Hospital of Stomatology, National Center for Stomatology, National Clinical Research Center for Oral Diseases, National Engineering Research Center of Oral Biomaterials and Digital Medical Devices, Beijing Key Laboratory of Digital Stomatology, Research Center of Engineering and Technology for Computerized Dentistry, Ministry of Health, NMPA Key Laboratory for Dental Materials, Beijing 100081, China.; ^2^College of Fisheries, Guangdong Ocean University, Zhanjiang 524088, Guangdong, China.; ^3^Center of Basic Medical Research, Institute of Medical Innovation and Research, Peking University Third Hospital, Beijing 100191, China.

**Keywords:** Angiogenesis, deferoxamine, stem cells from human exfoliated deciduous teeth, small extracellular vesicles, hydrogel

## Abstract

**Aim:** We investigated whether the secretome, particularly small extracellular vesicles (sEVs), obtained from deferoxamine (DFO)-pretreated stem cells from human exfoliated deciduous teeth (SHED), could promote angiogenesis in human umbilical vein endothelial cells (HUVECs) and support vascularized tissue integration under preliminary ectopic implantation conditions.

**Methods:** SHED were treated with DFO (2.5, 5, 10 μM), and RNA sequencing was performed to analyze gene expression changes in DFO-treated SHED. The effects of the DFO-pretreated SHED secretome and the isolated sEVs (DFO-sEVs) on HUVEC migration were evaluated. The angiogenic capacity was assessed using wound healing, Transwell migration, and tube formation assays. DFO-sEVs were also tested for their ability to regulate macrophage polarization using immunofluorescence staining. Finally, DFO-sEVs were encapsulated in hydrogels and subcutaneously implanted into Sprague-Dawley rats, and their pro-angiogenic capacity *in vivo* was assessed *via* histological staining.

**Results:** RNA-seq analysis indicated that DFO pretreatment modulated SHED pathways related to hypoxia response, cell adhesion, and inflammation. The secretome from SHED pretreated with 5 μM DFO significantly promoted HUVEC migration and tube formation. DFO-sEVs, particularly from the 5 μM DFO group, demonstrated superior pro-angiogenic effects compared to sEVs from untreated SHED both *in vitro* and *in vivo*. Furthermore, DFO-sEVs drove macrophages to adopt an M2-like reparative polarization.

**Conclusion:** DFO pretreatment functionally potentiates the pro-angiogenic and immunomodulatory properties of SHED-derived sEVs. The findings suggest that DFO-sEVs may provide a bioactive component for promoting angiogenesis and facilitating tissue integration.

## INTRODUCTION

Mesenchymal stem cells (MSCs) exhibit the capacity for multilineage differentiation and facilitate tissue repair primarily through paracrine mechanisms, supporting their broad application potential in regenerative medicine^[1,2]^. The MSC secretome captures the principal paracrine mediators of MSC activity and provides a cell-free therapeutic strategy with the potential to avoid limitations inherent to living-cell therapies^[1,2]^. Stem cells from human exfoliated deciduous teeth (SHED), as a dental pulp-originated MSC population, offer distinct advantages, including abundant source availability and minimal invasiveness in procurement compared to other MSC types^[3]^. The conditioned medium from SHED (SHED-CM) enhances the proliferative and metabolic activities of human umbilical vein endothelial cells (HUVECs) via its enriched vascular endothelial growth factor (VEGF) content^[4]^. However, the clinical translation of conditioned media (CM) remains limited by donor- and process-dependent compositional heterogeneity, poorly standardized concentrations and potency, batch-to-batch variability, downstream purification requirements, and storage-related alterations in bioactive components^[5,6]^.

The MSC secretome holds therapeutic promise that extends beyond its soluble components. In addition to proteins and growth factors, extracellular vesicles, represented by small extracellular vesicles (sEVs), constitute another major bioactive fraction of the stem cell secretome^[7]^. EVs are lipid-bilayer-delimited particles that carry bioactive cargo, including miRNAs, mRNAs, peptides, and proteins, and can alter the functions and phenotypes of recipient cells. When classified operationally by size, sEVs are generally described as EVs smaller than 200 nm, although no universally accepted size cut-off has been established^[8]^. Previous studies have revealed that SHED-derived sEVs promote bone defect repair by regulating angiogenesis and osteogenesis^[9,10]^. In an lipopolysaccharide (LPS)-stimulated murine skin wound model, SHED-derived sEVs carrying miR-1246 enhanced macrophage autophagy, thereby accelerating tissue regeneration and reducing pruritus^[11]^. Moreover, SHED-derived sEVs carry abundant anti-aging signals that alleviate the senescent phenotype exhibited by aged stem cells and preserve their tenogenic potential^[12]^.

The fate and function of stem cells are regulated by their culture microenvironment, and preconditioning approaches, including biochemical induction, physical stimulation, and genetic modification, can influence the paracrine profile of MSCs^[13]^. Our previous work demonstrated that sEVs derived from SHED cultured under hypoxic conditions (1% O_2_) exhibit enhanced pro-angiogenic and osteogenic capacity via activation of the VEGF signaling pathway^[10]^. Compared with hypoxic culture, chemical mimetics of hypoxia offer greater operational feasibility and consistency. Deferoxamine (DFO), an iron-chelating hypoxia mimetic, promotes hypoxia-inducible factor 1α (HIF-1α) stabilization and subsequently upregulates the expression of pro-angiogenic factors, including VEGF^[14]^. Siavashi *et al*. showed that DFO enhances the pro-angiogenic activity of the endothelial progenitor cell secretome, thereby improving tissue regeneration^[15]^. However, few reports currently describe the use of DFO preconditioning to obtain functionally optimized sEVs from stem cells.

In this work, we systematically investigated the angiogenic potential and immunomodulatory capacity of the sEVs derived from DFO-preconditioned SHED (DFO-SHED). We initially performed RNA sequencing on DFO-SHED. sEVs derived from DFO-SHED stimulated with varying DFO concentrations (DFO-sEVs) were subsequently isolated by ultracentrifugation. Using HUVECs and LPS-preconditioned RAW264.7 macrophages, we demonstrated that sEVs derived from 5 μM DFO-preconditioned SHED (DFO 5 μM-sEVs) significantly promoted HUVEC migration and tube formation, while enhancing the shift of RAW264.7 macrophages toward a pro-healing M2 phenotype. DFO-sEVs represent a promising cell-free bioactive component for the clinical management of chronic wounds characterized by impaired angiogenesis and excessive inflammation.

## METHODS

### Cell culture

SHED were obtained from the ORAL STEM CELL BANK operated by Beijing Tason Biotech Co., Ltd. (Beijing, China), with an internal lot number of MC-BJ170900060. Cells were resuspended into a single-cell suspension and maintained in Minimum Essential Medium Alpha (α-MEM, Gibco, Thermo Fisher Scientific, USA; Cat. No. C12571500BT) containing 10% fetal bovine serum (FBS, ABW, China; Cat. No. AB-FBS-1050S) and 1% penicillin-streptomycin. Cells were cultured at 37 °C in a humidified atmosphere containing 5% CO_2_ and were used for experiments at passages 3-7.

HUVECs were obtained from Chi Scientific Company (China; Cat. No. 7-1074); Short Tandem Repeat profiling for these cells was provided by the supplier. HUVECs were maintained in M199 medium (Gibco, Thermo Fisher Scientific, USA; Cat. No. C11150500BT) containing 10% FBS and 1% penicillin-streptomycin and used for experiments at passages 4-6.

For both SHED and HUVEC experiments, cells at the same passage were used across all experimental groups within each independent experiment. All cells were routinely screened for mycoplasma contamination immediately after recovery from cryopreservation and prior to each experiment. Detection was carried out using a Mycoplasma Rapid Detection Kit (ABclonal; Cat. No. BRK0043).

### Stem cell identification

#### Flow cytometric analysis

Passage 3 SHED were harvested for flow cytometric analysis of cell-surface markers. Harvested cells were transferred to flow cytometry tubes for centrifugation (300 × *g*, 5 min). After discarding the supernatant, the resulting pellets were rinsed twice with pre-cooled phosphate-buffered saline (PBS) and subsequently resuspended in 200 μL PBS. Fluorochrome-conjugated antibodies were added to the cell suspension: Allophycocyanin Mouse Anti-Human CD29 (APC Mouse Anti-Human CD29, BD Pharmingen, USA; Cat. No. 559883), phycoerythrin Mouse Anti-Human CD73 (PE Mouse Anti-Human CD73, BD Pharmingen, USA; Cat. No. 550257), and fluorescein isothiocyanate Mouse Anti-Human CD45 (FITC Mouse Anti-Human CD45, BD Pharmingen, USA; Cat. No. 555482). The antibody incubation was carried out at room temperature for 1 h in the dark. After antibody labeling, a single PBS wash was performed before the cell suspension was filtered through a cell strainer. Flow cytometric acquisition was performed using appropriate detection channels based on each fluorophore’s emission wavelength. Unstained cells served as controls for instrument calibration and negative gating.

#### Osteogenic differentiation

SHED at passage 3 were seeded into 12-well plates and allowed to reach approximately 80% confluence. Osteogenic induction was initiated by washing the cells with PBS three times and switching to the induction medium. The induction medium was renewed at 3-day intervals, and induction continued for 14 days. For Alizarin Red S (ARS) staining, cells subjected to osteogenic induction were fixed in 4% paraformaldehyde for 30 min at room temperature. ARS working solution was applied to the cells for 30 min. Excess staining solution was removed by repeated rinsing in distilled water, followed by optical microscopy to examine the mineralized nodules.

### Adipogenic differentiation

SHED at passage 3 were seeded into 12-well plates and cultured until approximately 80% confluence was reached. After three PBS washes, the cells were switched to adipogenic induction medium. The induction medium was supplied at 2-day intervals for 21 days. Following adipogenic induction, lipid accumulation was assessed by Oil Red O (ORO) staining. Fixation was carried out with 4% paraformaldehyde for 30 min at room temperature. The staining working solution was applied to the cells for 30 min. After removing excess stain, lipid droplets were visualized using an optical microscope.

### Collection of SHED-derived conditioned medium

DFO (Sigma-Aldrich, USA, Cat. No. D9533-1G, Lot No. BCCN3164) was dissolved in sterile water to prepare a 50 mM stock solution following the manufacturer’s instructions. Immediately before use, the stock solution was serially diluted with sterile water to working concentrations of 250 μM, 500 μM, 1 mM, 2 mM, 5 mM, 10 mM, and 20 mM. Each working solution was mixed with cell culture medium at a 1:99 (v/v) dilution ratio to generate DFO-culture medium at 2.5, 5, 10, 20, 50, 100, and 200 μM, respectively.

SHED at passages 4-7 were left untreated or subjected to DFO pretreatment at 2.5, 5, or 10 µM for 48 h. After extensive rinsing with PBS to remove residual DFO, the medium was replaced with DFO-free medium. After another 24 h, the conditioned medium was harvested for subsequent CM experiments or sEV isolation.

In parallel, cell-free culture medium was incubated under the same culture conditions for 24 h and collected as a blank control. All collected media were centrifuged (3,000 rpm, 10 min) to eliminate cell debris. The resulting media were mixed with an equal volume of serum-free medium and designated as Blank, Ctrl-CM, DFO 2.5 µM-CM, DFO 5 µM-CM, and DFO 10 µM-CM, respectively.

### Isolation of SHED-derived sEVs

Conditioned medium collected from SHED at passages 4-7 was used for sEV isolation. A sequential centrifugation procedure was conducted at 4 °C, consisting of an initial centrifugation at 300 × *g* for 10 min, followed by 16,500 × *g* for 20 min. The supernatant was then concentrated using a 100 kDa ultrafiltration centrifuge tube at 5,000 × *g* for 10 min. The concentrated sample was passed through a 0.22 μm membrane and transferred to ultracentrifuge tubes. sEVs were collected by centrifugation at 120,000 × *g* for 70 min, washed with PBS, and centrifuged again under the same conditions. After resuspension in 50 μL of PBS, the pellet was stored at -80 °C.

### Characterization of SHED-derived sEVs

Morphology: An sEV suspension (10 µL) was deposited onto a copper grid, then blotted with filter paper after 5 min. The grid was negatively stained with 10 μL of 1% phosphotungstic acid for 10 min, air-dried, and observed under transmission electron microscopy (TEM).

Nanoparticle tracking analysis (NTA) for Size: sEV samples were appropriately diluted with PBS and analyzed using an NTA instrument to determine particle size distribution.

Western Blot Analysis for Marker Proteins: Expression of sEV-associated markers CD9 (Abcam, USA; Cat. No. ab263019, RRID: AB_3076464) and TSG101 (Abcam, USA; Cat. No. ab125011, RRID: AB_10974262) was examined using Western blotting, with Calnexin (Abcam, USA; Cat. No. ab133615, RRID: AB_2864299) included as a negative control (cell lysate marker). Western blot experiments were performed using three independent biological replicates (*n* = 3), with three technical replicates for each biological replicate.

All characterizations were conducted using three independent biological replicates (*n* = 3), and each replicate consisted of sEV samples from all experimental groups prepared within the same experimental batch.

### Cell proliferation assay (CCK-8)

SHED at passages 3-6 were plated into 96-well plates at 2 × 10^3^ cells/well. After overnight culture, the medium was exchanged for DFO-medium (2.5, 5, 10, 20, 50, 100, and 200 μM). Untreated SHED cultured without DFO (0 μM DFO) served as the cell control group (Ctrl). Wells containing culture medium without cells were included as blank controls for background correction. Cell activity was evaluated on days 1, 4, and 7 after DFO treatment using a Cell Counting Kit-8 (CCK-8, Beyotime, China; Cat. No. C0038). At each time point, the cells were treated with 110 µL of working solution (10 µL CCK-8 reagent + 100 µL fresh medium). After 1 h of incubation (37 °C, dark conditions), absorbance was measured using a microplate reader (450 nm).

### Live-dead assay

The cytotoxic effects of DFO were assessed by live/dead staining (Beyotime, China; C2015S). SHED at passages 4-7 were seeded in 24-well plates and pretreated with DFO (2.5, 5, or 10 μM) for 48 h. After PBS washing, the staining solution was generated by diluting Calcein AM and PI stock solutions (both 1,000×) at a 1:1,000 ratio in detection buffer. After addition of the staining solution, the cells were maintained at 37 °C for 40 min under light-protected conditions and then observed with an Olympus fluorescence microscope. Each group included three independent biological replicates, and representative images were selected for presentation.

### Cell migration

#### Wound healing

SHED at passages 3-6 were seeded in 12-well plates (1 × 10^5^ cells per well). After achieving 100% confluence, a linear scratch was generated across the cell monolayer using a 200 μL tip. The cells were maintained in low-serum medium containing 1% FBS alone (Ctrl) or supplemented with DFO at concentrations of 2.5, 5, or 10 µM. After 10 h of incubation, wound-closure images were acquired under an optical microscope.

HUVECs at passages 4-6 were seeded in 12-well plates (1 × 10^5^ cells per well). After achieving 100% confluence, a linear scratch was generated across the cell monolayer using a 200 μL tip. The cells were incubated with the collected media preparations described in Section “Collection of SHED-derived conditioned medium”: Blank, Ctrl-CM, DFO 2.5 µM-CM, DFO 5 µM-CM, and DFO 10 µM-CM. After 12 h of incubation, images were acquired under an optical microscope.

HUVECs at passages 4-6 were seeded in 12-well plates (1 × 10^5^ cells per well). After achieving 100% confluence, a linear scratch was generated across the cell monolayer using a 200 μL tip. The cells were maintained in low-serum medium containing 1% FBS without sEVs (Ctrl) or supplemented with 10 µg/mL sEVs, 10 µg/mL DFO 2.5 µM-sEVs, 10 µg/mL DFO 5 µM-sEVs, or 10 µg/mL DFO 10 µM-sEVs. After 12 h of incubation, images were acquired under an optical microscope. Micrographs were analyzed using ImageJ software.

#### Transwell migration assay

A HUVEC suspension (passages 4-6, 200 µL containing 1 × 10^4^ cells) was introduced into the Transwell (8 µm pore size, Corning) upper compartment. The lower compartments were supplemented with 600 µL of different CM preparations (prepared as described in Section “Collection of SHED-derived conditioned medium”), including Blank, Ctrl-CM, DFO 2.5 µM-CM, DFO 5 µM-CM, and DFO 10 µM-CM; or with 600 µL of low-serum medium (1% FBS) alone (Ctrl) or containing 10 µg/mL sEVs, 10 µg/mL DFO 2.5 µM-sEVs, 10 µg/mL DFO 5 µM-sEVs, or 10 µg/mL DFO 10 µM-sEVs. After 24 h, non-migrated cells were gently removed. After PBS washing, the inserts underwent fixation with 4% paraformaldehyde for 15 min, followed by staining with 1% crystal violet for 30 min. Images of migrated cells from five random fields were acquired. Micrographs were analyzed using ImageJ software.

### *In vitro* angiogenesis

For DFO-pretreated SHED-CM, Matrigel (50 µL per well, Corning, USA; Cat. No. 356234) was polymerized in 96-well plates at 37 °C for 1 h. HUVECs at passages 4-6 were suspended in different collected media (prepared as described in Section “Collection of SHED-derived conditioned medium”), including Blank, Ctrl-CM, DFO 2.5 µM-CM, DFO 5 µM-CM, and DFO 10 µM-CM. The prepared cell suspensions were transferred onto Matrigel at 2.5 × 10^4^ cells per well. Following a 6 h incubation period, tube formation was examined using an inverted microscope.

To assess the pro-angiogenic effect of sEVs derived from DFO-pretreated SHED, Matrigel (50 µL per well, Corning) was polymerized in 96-well plates at 37 °C for 1 h. HUVECs at passages 4-6 were resuspended in low-serum medium (1% FBS) without sEVs (Ctrl) or in low-serum medium supplemented with 10 µg/mL sEVs, 10 µg/mL DFO 2.5 µM-sEVs, 10 µg/mL DFO 5 µM-sEVs, or 10 µg/mL DFO 10 µM-sEVs. The prepared cell suspensions were transferred onto Matrigel at 2.5 × 10^4^ cells per well. Following a 6 h incubation period, tube formation was examined using an inverted microscope. Micrographs were analyzed using ImageJ software.

### Detection of macrophage polarization phenotypes by immunofluorescence staining

For immunofluorescence staining, RAW264.7 cells were plated in confocal dishes (5 × 10^4^ cells/well) and stimulated using 100 ng/mL LPS for 24 h. Cells maintained in standard medium without LPS served as the negative control (Ctrl), whereas cells treated with LPS before the medium change constituted the positive control [LPS + Dulbecco’s Modified Eagle Medium (DMEM)]. The experimental groups comprised LPS + sEVs, LPS + DFO 2.5 µM-sEVs, LPS + DFO 5 µM-sEVs, and LPS + DFO 10 µM-sEVs. After three days of treatment, the cells were washed, fixed, permeabilized, and blocked before incubation with inducible Nitric Oxide Synthase polyclonal antibody (iNOS polyclonal antibody,Proteintech, China, Cat. No. 18985-1-AP) and CD206 monoclonal antibody (Proteintech, China, Cat. No. 60143-1-Ig). Fluorescence images were acquired by CLSM and analyzed using ImageJ software.

### Subcutaneous implantation model in Sprague-Dawley rats

DFO-sEVs refer to sEVs derived from SHED preconditioned with 5 μM DFO, which was selected based on *in vitro* optimization for subsequent *in vivo* use.

All animal experiments were conducted in accordance with the regulations of the Tianjin Medical Experimental Animal Care. The experimental protocols were approved by the Institutional Animal Care and Use Committee (IACUC) of Yi Shengyuan Gene Technology (Tianjin) Co., Ltd. (protocol number: YSY-DWLL-2025952).

Six male Sprague-Dawley (SD) rats (6 weeks old; approximately 280 g) were used to establish the ectopic subcutaneous implantation model. Bilateral dorsal subcutaneous pockets were created under sterile conditions using inhalational isoflurane anesthesia. GelMA, sEVs@GelMA, and DFO-sEVs@GelMA scaffolds (1 cm in diameter) were randomly allocated and implanted into the subcutaneous pockets. Each scaffold type was implanted at four independent sites. Scaffold preparation and implantation were performed by two independent investigators. The operator responsible for implantation was blinded to scaffold coding during the procedure.

One week after implantation, rats were euthanized by CO_2_ inhalation, and tissue specimens were harvested and formalin-fixed at 10% concentration. The specimens were processed through sequential ethanol dehydration and paraffin embedding. Each specimen was sectioned through its central region at a thickness of 5 μm, and the sections were subjected to Hematoxylin-Eosin (HE) Stain Kit (Solarbio, China; Cat. No. G1120), Masson’s Trichrome Staining Kit (Baso, China; Cat. No. BA4079B), and CD31 immunohistochemistry (Abcam, USA; Cat. No. ab182981, RRID: AB_2920881) following standard protocols. All histological sections were examined using light microscopy.

### RNA sequencing and bioinformatics analysis

Passage 5 SHED were used for RNA sequencing. SHED samples underwent total RNA extraction using the miRNeasy Micro Kit (Qiagen). RNA purity (A260/A280 ratio of 1.8-2.1) and concentration were determined using a NanoDrop spectrophotometer, and RNA integrity was evaluated by the LabChip system (RIN value). All samples had RIN values ≥ 8.3, meeting the requirements for library construction. Three biological replicates were included per group (control group: ctl-1, ctl-2, ctl-3; treatment group: DFO-1, DFO-2, DFO-3).

RNA libraries were prepared with the VAHTS® Universal V8 RNA-seq Library Prep Kit for Illumina and sequenced using an Illumina NovaSeq 6000 platform (PE150). Raw sequencing reads were processed with Cutadapt (v1.9.1) to eliminate low-quality sequences and adapter contamination. Clean reads were mapped against the human reference genome GRCh38 (Ensembl release 107) using HISAT2 (v2.2.1).

Gene expression profiles were quantified with HTSeq (v0.6.1). Differentially expressed genes (DEGs) were identified using DESeq2 (v1.26.0) based on the negative binomial distribution and selected according to the criteria of |log_2_(fold change)| ≥ log_2_(1.5) and *Q*-value < 0.05. The *Q*-values were adjusted using the Benjamini-Hochberg correction method.

Gene Ontology (GO) enrichment analysis was conducted with GOseq based on the Wallenius non-central hypergeometric distribution, with *P*-value ≤ 0.05 considered statistically significant. Kyoto Encyclopedia of Genes and Genomes (KEGG) pathway enrichment analysis was assessed using a hypergeometric test, and pathways with *P*-value ≤ 0.05 were regarded as significantly enriched.

The RNA-seq datasets generated in this study are available in the SRA under BioProject accession number PRJNA1512113.

### Statistical analysis

Statistical analysis was conducted with IBM SPSS Statistics 26.0 software (IBM Corp., Armonk, NY, USA). Quantitative results are presented as mean ± SD from at least three biological replicates (*n* ≥ 3). The data were first assessed for normality and homogeneity of variance, after which statistical analyses were carried out. For the CCK-8 assay, the effects of DFO concentration, incubation duration, and their interaction were evaluated using two-way analysis of variance (ANOVA), with subsequent Tukey’s multiple comparison test. In contrast, other multi-group comparisons were assessed using one-way ANOVA, and Tukey’s honestly significant difference (HSD) test was subsequently applied for post hoc analysis. Differences were considered statistically significant when *P* < 0.05.

## RESULTS

### Effects of DFO preconditioning on SHED and their secretome

The mycoplasma test results showed that the SHED sample remained red after incubation, indicating a negative result for mycoplasma contamination [Supplementary Figure 1].

SHED exhibited a typical spindle-shaped fibroblast-like morphology under an inverted microscope [Supplementary Figure 2A]. Osteogenic and adipogenic differentiation assays demonstrated positive ARS and ORO staining, respectively [Supplementary Figure 2B and C]. Flow cytometry demonstrated a CD29^+^/CD73^+^/CD45^-^ phenotype in SHED [Supplementary Figure 2D], confirming the MSC characteristics of the isolated cells.

To assess DFO effects on SHED viability, a CCK-8 assay together with live/dead staining was performed. 2.5-10 µM DFO did not reduce the metabolic activity of SHED, whereas 20 µM DFO significantly inhibited cell proliferation [Figure 1A]. Consistently, live/dead staining showed that cells in the Ctrl and 2.5, 5, and 10 µM DFO groups were predominantly viable, as indicated by strong green fluorescence, with only sparse red fluorescence signals corresponding to dead cells [Supplementary Figure 3]. These results suggest that 2.5-10 µM DFO did not induce substantial cell death under the present experimental conditions. Therefore, subsequent experiments used DFO at 2.5, 5, and 10 μM. As shown in Figure 1B and C, low concentrations of DFO (2.5 and 5 µM) significantly promoted SHED migration, with 5 µM DFO yielding the largest migration area. The 10 µM DFO group showed a decreasing trend in SHED migration, although this difference was not statistically significant when compared with the Ctrl group.

**Figure 1:**
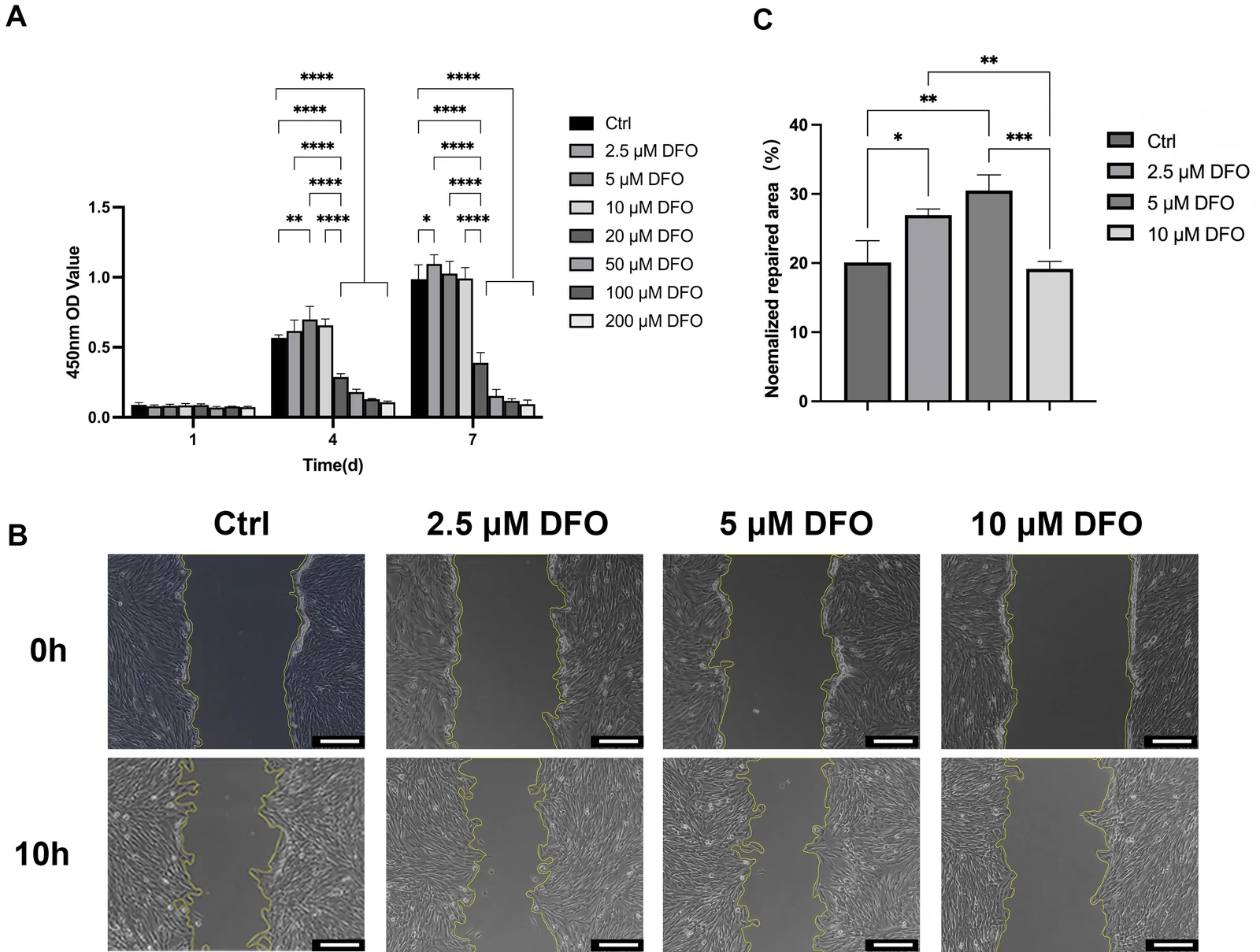
Responses of SHED to DFO Preconditioning. (A) Assessment of cell activity on days 1, 4, and 7. Data were analyzed by two-way ANOVA with Tukey’s multiple-comparisons test; four independent biological replicates were included (*n* = 4); (B) SHED migration; Scale bar = 200 μm; (C) quantification of the migration results. Data were analyzed by one-way ANOVA with Tukey’s HSD post hoc comparisons; three independent biological replicates were included (*n* = 3). ^*^P < 0.05; ^**^P < 0.01; ^***^P < 0.001; ^****^P < 0.0001. ANOVA: Analysis of variance; Ctrl: control; DFO: deferoxamine; HSD: honestly significant difference; OD: optical density; SHED: stem cells from human exfoliated deciduous teeth.

To investigate the transcriptomic landscape of SHED in response to DFO treatment, RNA sequencing was performed on SHED treated with 5 µM DFO, enabling the identification of DEGs between DFO-treated and untreated SHED [Figure 2A and B]. GO and KEGG analyses were further used to explore associated signaling pathways [Figure 2C and D]. Compared to the control, 247 genes were downregulated, and 159 genes were upregulated in DFO-treated SHED. GO enrichment analysis identified significant enrichment of “transferrin receptor activity” in the Molecular Function category, suggesting that DFO may affect cellular iron metabolism. The KEGG results showed that the “ferroptosis” pathway was enriched, indicating a potential association between DFO treatment and iron-dependent cell death regulation. As a classic hypoxia mimetic, DFO was associated with hypoxia through the “cellular response to hypoxia” pathway. Both the “TNF signaling pathway” and “Toll-like receptor signaling pathway” showed enrichment, further suggesting that DFO may also modulate inflammatory and anti-inflammatory responses.

**Figure 2:**
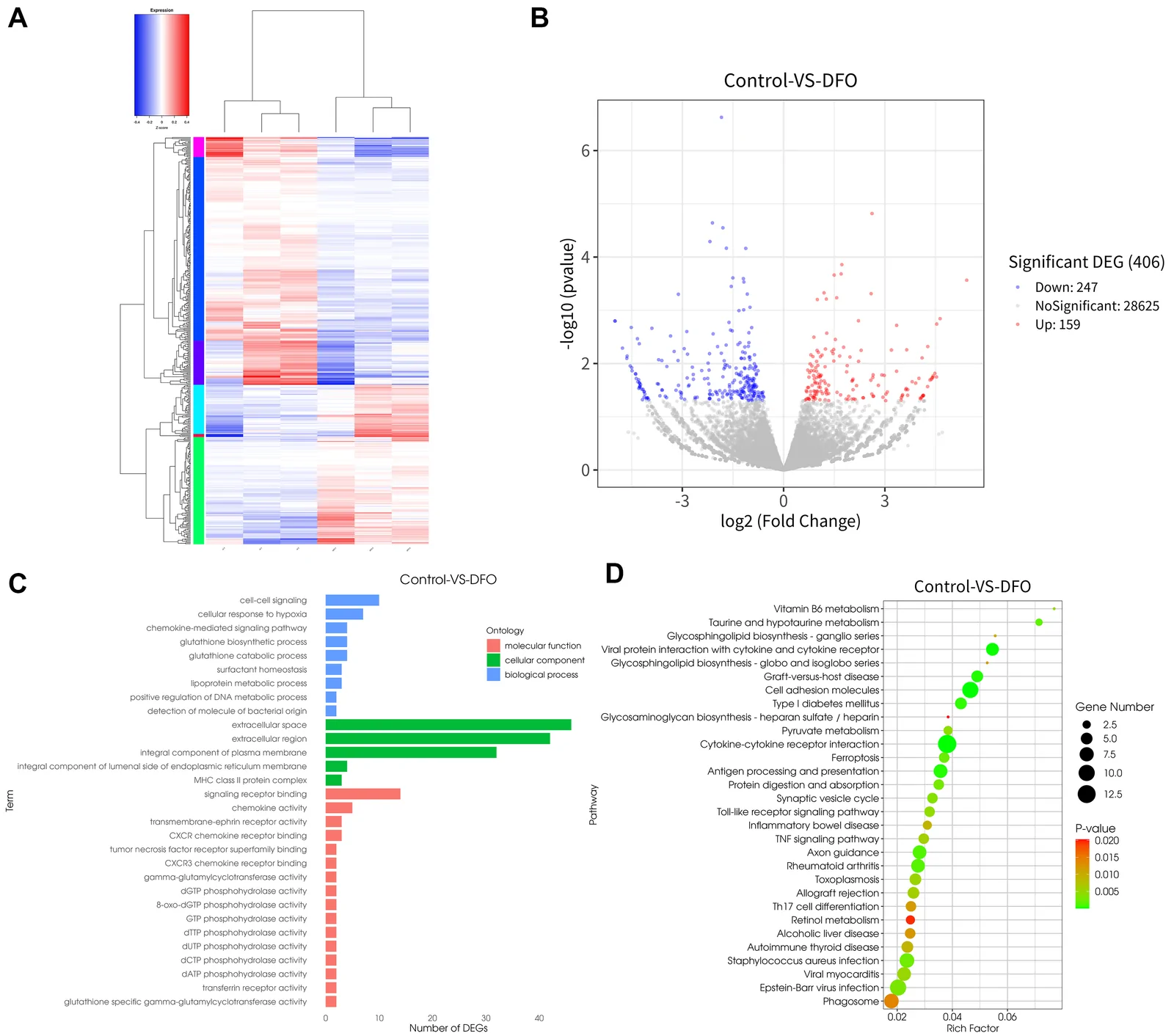
Transcriptomic Profiling Analysis. (A) Heatmap of clustering analysis; (B) volcano plot illustrating DEGs; (C) bar chart showing GO enrichment results; (D) KEGG enrichment results presented as a bubble plot. For RNA-seq, each group comprised three independent biological replicates (*n* = 3). Each replicate represents an RNA sample independently extracted from separately cultured SHED samples. ANOVA: Analysis of variance; CXCR: C-X-C chemokine receptor; CXCR3: C-X-C chemokine receptor 3; DEG: differentially expressed gene; DEGs: differentially expressed genes; DFC: deferoxamine-free control; DFO: deferoxamine; dATP: deoxyadenosine triphosphate; dCTP: deoxycytidine triphosphate; dGTP: deoxyguanosine triphosphate; dTTP: deoxythymidine triphosphate; dUTP: deoxyuridine triphosphate; GTP: guanosine triphosphate; GO: Gene Ontology; KEGG: Kyoto Encyclopedia of Genes and Genomes; MHC: major histocompatibility complex; RNA-seq: RNA sequencing; SHED: stem cells from human exfoliated deciduous teeth; TNF: tumor necrosis factor.

The mycoplasma test results showed that the HUVEC sample remained red after incubation, indicating a negative result for mycoplasma contamination [Supplementary Figure 1].

To assess the influence of CM from SHED treated with different DFO concentrations (2.5, 5, 10 µM) on HUVEC migration, scratch and Transwell assays were used. The study groups included a Blank group (serum-containing medium without cell culture), a Control group (CM from SHED without DFO treatment), and Treatment groups (CM from SHED treated with 2.5, 5, or 10 µM DFO). DFO-treated groups showed greater migration-promoting effects compared to both the Blank and Ctrl groups. Specifically, the normalized wound closure area was highest in the DFO 5 µM-CM group, followed by the DFO 10 µM-CM and DFO 2.5 µM-CM groups. Meanwhile, the number of migrated cells per field was highest in the DFO 2.5 µM-CM group, followed by the DFO 5 µM-CM and DFO 10 µM-CM groups [Supplementary Figures 4 and 5]. These findings suggest that CM from DFO-treated SHED promotes HUVEC migration. In the tube formation assay, the number of main junctions and total master segment length were higher in all DFO-CM groups compared to the Blank and Ctrl groups. Specifically, the DFO 2.5 µM-CM and DFO 5 µM-CM groups showed comparable and greater tube formation parameters than the DFO 10 µM-CM group [Supplementary Figure 6], indicating that CM from DFO-treated SHED enhances HUVEC angiogenesis.

### Isolation and characterization of sEVs derived from DFO-treated SHED

sEVs are key mediators of intercellular communication within the SHED secretome. sEVs were isolated from CM of SHED treated with 0, 2.5, 5, or 10 µM DFO via ultracentrifugation and designated as sEVs, DFO 2.5 µM-sEVs, DFO 5 µM-sEVs, and DFO 10 µM-sEVs, respectively. TEM analysis demonstrated that all sEVs displayed a characteristic cup-shaped morphology with an identifiable bilayer membrane structure [Figure 3A]. NTA showed similar size distributions among all groups. The mean particle diameters of sEVs, DFO 2.5 µM-sEVs, DFO 5 μM-sEVs, and DFO 10 μM-sEVs were within the range of 100-150 nm, indicating that DFO pretreatment did not significantly alter the particle size of SHED-derived sEVs [Figure 3B]. Western blotting detected the sEV-associated markers TSG101 and CD9, whereas the cellular marker Calnexin was not detected [Figure 3C]. Although the CD9 signal appeared reduced in DFO-treated groups, equal amounts of total sEV protein were loaded for Western blot analysis. Therefore, CD9 intensity alone may not directly reflect total sEV abundance or functional potency. The isolated vesicles retained typical sEV morphology and size distribution and expressed other sEV-associated markers, suggesting that the reduced CD9 signal may reflect DFO-induced changes in surface marker enrichment, cargo sorting, or sEV subpopulation composition rather than a loss of overall sEV identity.

**Figure 3:**
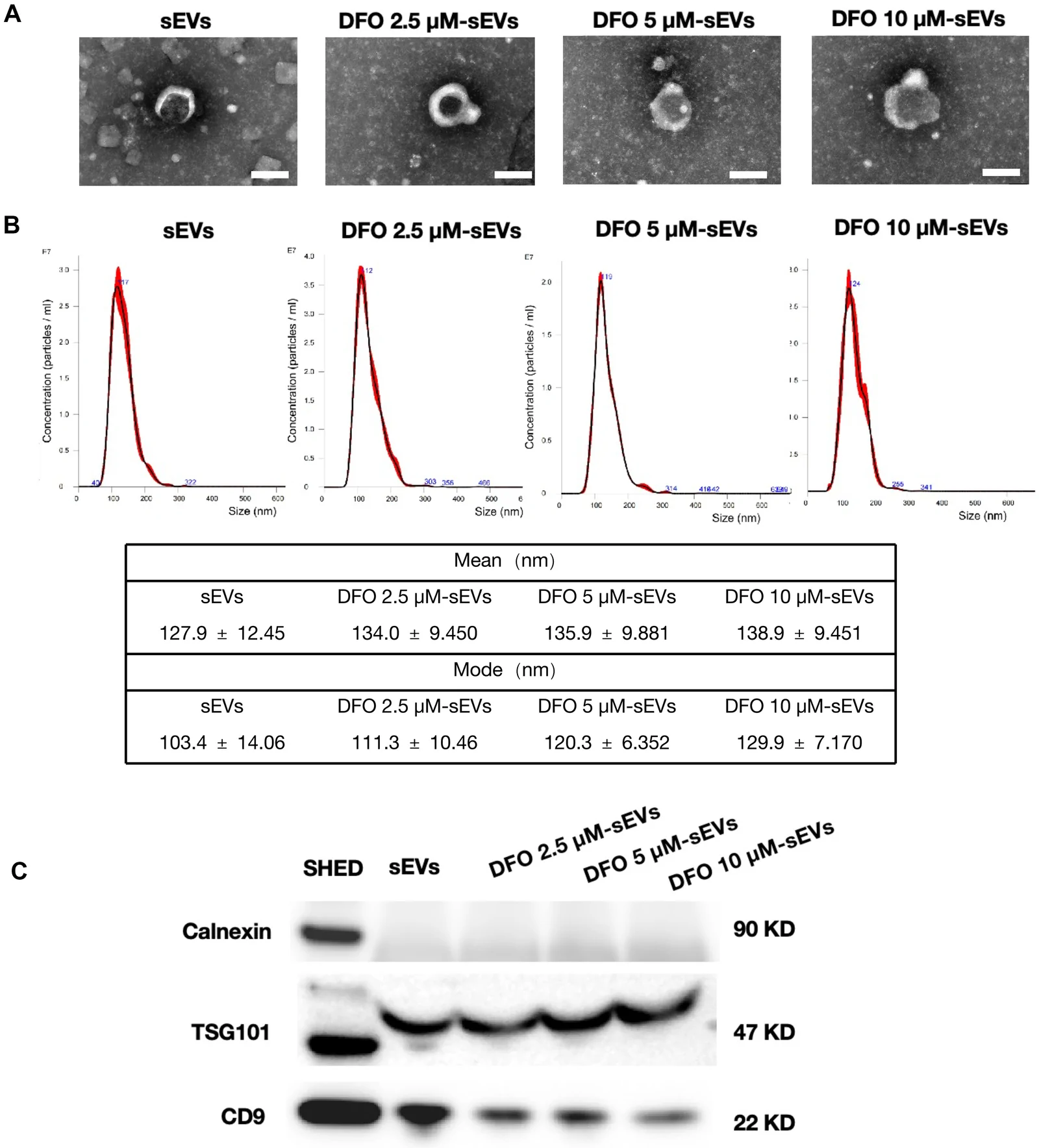
Characterization of SHED-derived sEVs. (A) sEV morphology observed by TEM; Scale bar = 100 nm; (B) size distribution of sEVs determined by NTA. Representative size distribution profiles are shown. The mean and mode particle sizes were calculated from three independent sEV preparations and are expressed as the mean ± SD (*n* = 3); (C) Western blot analysis of sEV-associated markers. For Western blot analysis, three technical replicates were performed for each biological replicate. All experiments included three independent biological replicates. Representative images are shown. CD9: Cluster of differentiation 9; DFO: deferoxamine; KD: kilodalton; NTA: nanoparticle tracking analysis; sEVs: small extracellular vesicles; SD: standard deviation; SHED: stem cells from human exfoliated deciduous teeth; TEM: transmission electron microscopy; TSG101: tumor susceptibility gene 101.

### Effects of DFO-sEVs on HUVEC migration and angiogenesis

To assess the influence of various sEV treatments on HUVEC migration, scratch and Transwell assays were performed using sEVs (10 µg/mL) added to low-serum medium [Figure 4A and B]. The DFO 5 µM-sEVs group exhibited the most significant pro-migratory effect, with a normalized wound closure area of 55.44% (1.29-fold higher than Ctrl, *P* < 0.0001; 1.15-fold higher than sEVs, *P* < 0.001, *n* = 3) and 99 migrated cells per field (1.91-fold higher than Ctrl, *P* < 0.0001; 1.37-fold higher than sEVs, *P* < 0.0001), indicating that DFO 5 µM-sEVs significantly promoted both horizontal and vertical migration of HUVECs. Subsequent tube formation assays on Matrigel demonstrated that, relative to the Control and sEVs groups, all sEV-treated groups promoted greater angiogenesis. Specifically, the DFO 5 µM-sEVs and DFO 10 µM-sEVs groups showed significantly higher numbers of main junctions and total master segment length relative to the sEVs group (*P* < 0.05, *n* = 3) [Figure 4C and D].

**Figure 4:**
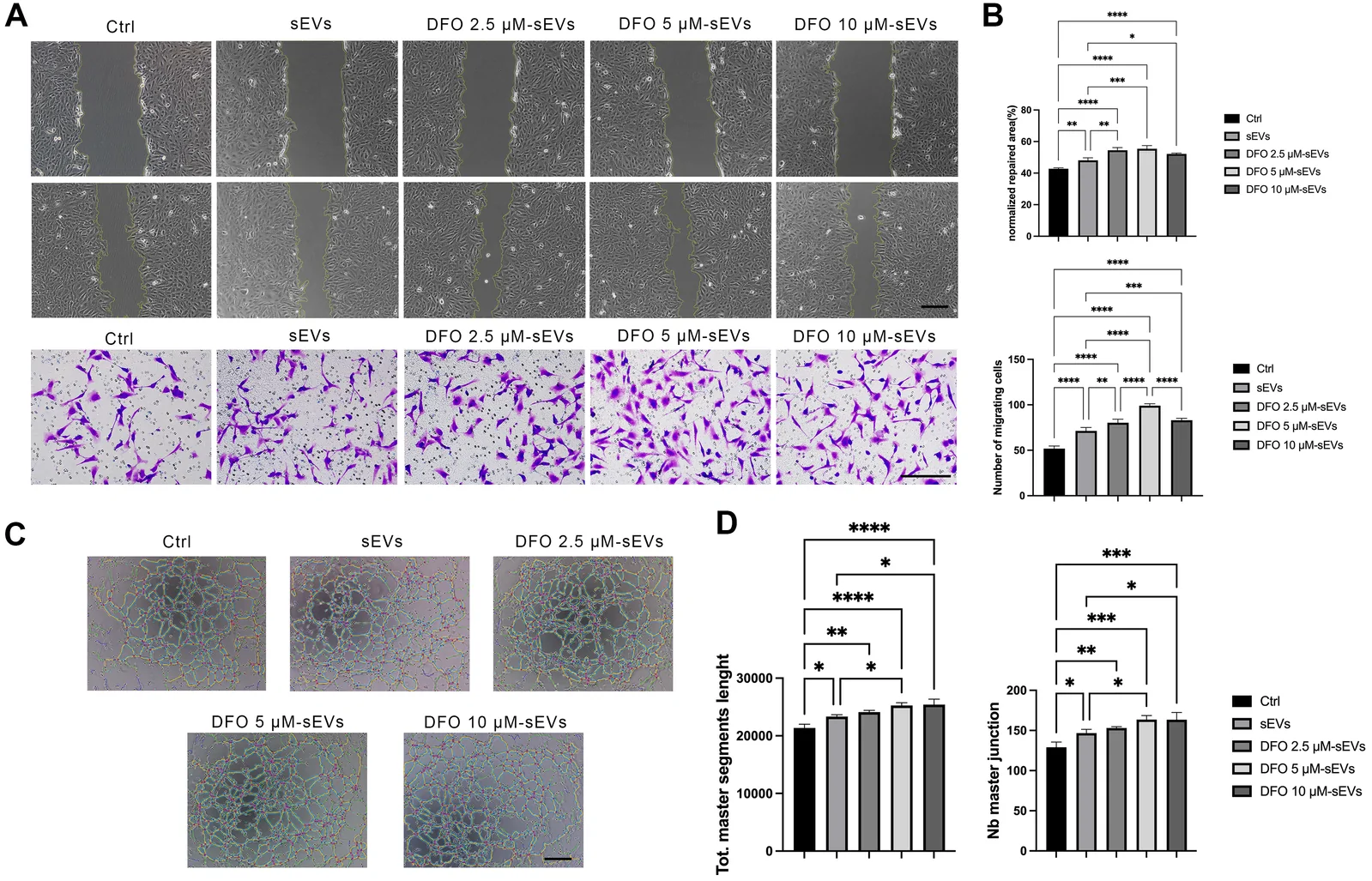
Effects of DFO-sEVs on HUVECs migration and angiogenesis. (A) HUVEC migration assessed using wound-healing and Transwell assays; Scale bar = 200 μm, with (B) quantification of migration; (C) HUVEC tube formation *in vitro*; Scale bar = 200 μm; (D) quantification of total master segment length and number of master junctions. Values are expressed as mean ± SD. Data were analyzed by one-way ANOVA with Tukey’s HSD post hoc comparisons; at least three independent biological replicates were included. ^*^P < 0.05; ^**^P < 0.01; ^***^P < 0.001; ^****^P < 0.0001. ANOVA: Analysis of variance; Ctrl: control; DFO: deferoxamine; HSD: honestly significant difference; HUVECs: human umbilical vein endothelial cells; Nb: number; SD: standard deviation; sEVs: small extracellular vesicles; Tot.: total.

### Regulation of macrophage polarization by DFO-sEVs

To assess how DFO-sEVs modulate pro-inflammatory macrophage phenotype and function, an M1 macrophage phenotype was first induced using LPS, followed by exposure to different sEV preparations. Immunofluorescence staining revealed that, compared to the Ctrl group, LPS-stimulated macrophages showed the highest iNOS-associated green fluorescence, whereas CD206 staining remained largely negative, suggesting successful M1 macrophage activation. The addition of sEVs significantly reduced LPS-induced M1 activation, as shown by decreased iNOS expression [Figure 5A] and increased CD206 expression [Figure 5B]. The anti-inflammatory potency of different sEV groups followed this order: DFO 5 µM-sEVs > DFO 2.5 µM-sEVs ≈ DFO 10 µM-sEVs > sEVs.

**Figure 5:**
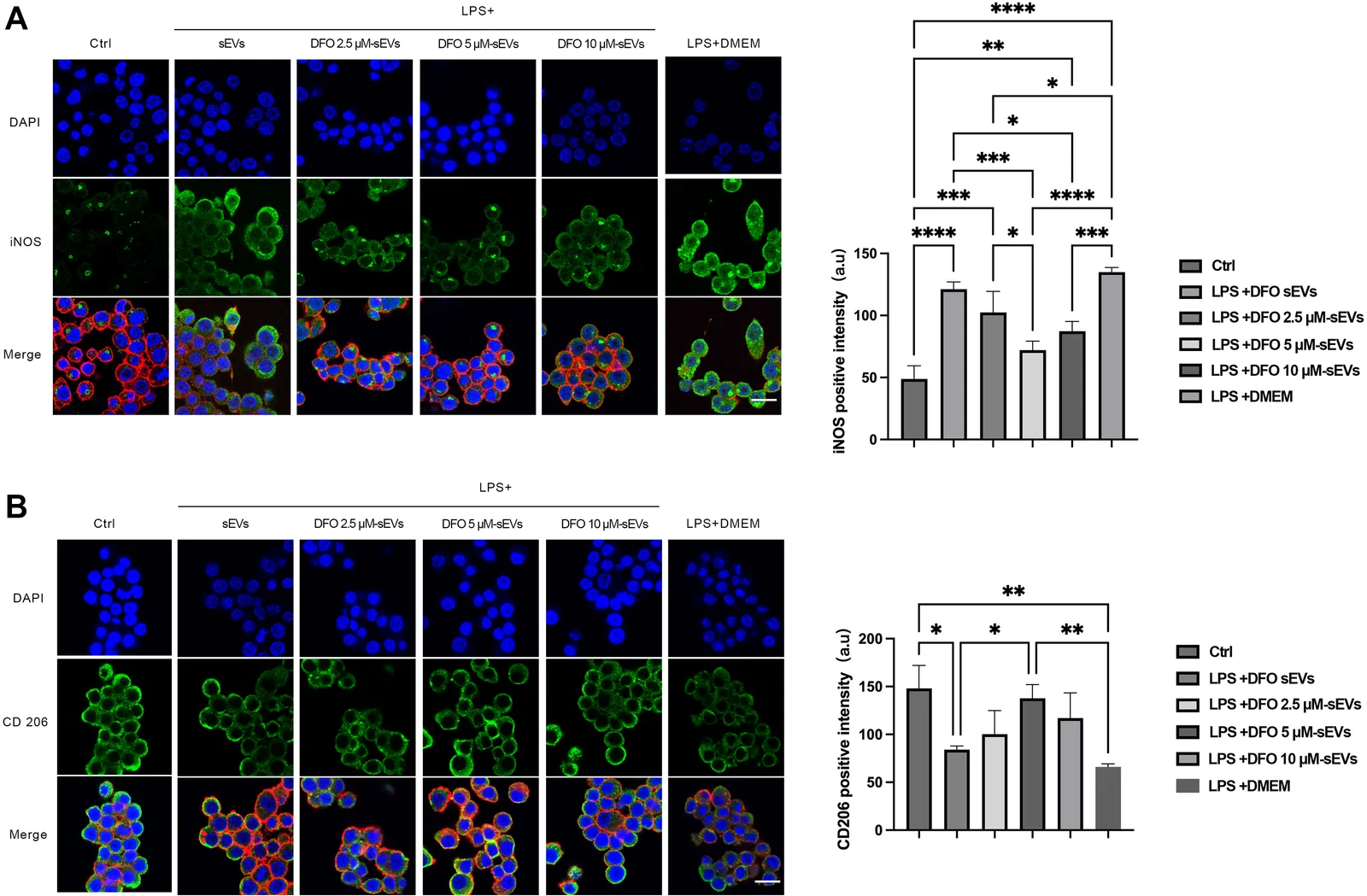
Modulatory effects of DFO-sEVs on RAW264.7 cell polarization. (A) iNOS expression on day 3; Scale bar = 20 μm, with quantification of immunofluorescence intensity; (B) CD206 expression on day 3; Scale bar = 20 μm, with quantification of immunofluorescence intensity. Values are expressed as mean ± SD. Data were analyzed by one-way ANOVA with Tukey’s HSD post hoc comparisons; three independent biological replicates were included. ^*^P < 0.05; ^**^P < 0.01; ^***^P < 0.001; ^****^P < 0.0001. ANOVA: Analysis of variance; a.u.: arbitrary units; CD206: cluster of differentiation 206; Ctrl: control; DAPI: 4′,6-diamidino-2-phenylindole; DFO: deferoxamine; DMEM: Dulbecco’s modified Eagle medium; HSD: honestly significant difference; iNOS: inducible nitric oxide synthase; LPS: lipopolysaccharide; RAW264.7: murine macrophage cell line; SD: standard deviation; sEVs: small extracellular vesicles.

### Subcutaneous implantation of DFO-sEVs-loaded GelMA hydrogel achieves ectopic vascularization

GelMA, sEVs@GelMA, and DFO-sEVs@GelMA hydrogels were subcutaneously implanted in an ectopic model [Figure 6]. Histological sections of the material-only group revealed gaps between the residual hydrogel and the host tissue, indicating poor integration of GelMA in the absence of bioactive components [Figure 6B and C]. In contrast, the sEVs- and DFO-sEVs-loaded groups demonstrated tight integration with the surrounding tissue [Figure 6F, G, J, and K]. Immunohistochemical staining for CD31 was used to assess vascularization [Figure 6D, H, and L]. CD31 expression was weak in the GelMA group. Both the sEVs@GelMA and DFO-sEVs@GelMA groups exhibited strong CD31 positivity. Based on hematoxylin and eosin staining and the pattern of CD31 expression, the newly formed vessels in the sEVs@GelMA group were of smaller diameter, whereas the DFO-sEVs@GelMA group contained some larger neo-vessels.

**Figure 6:**
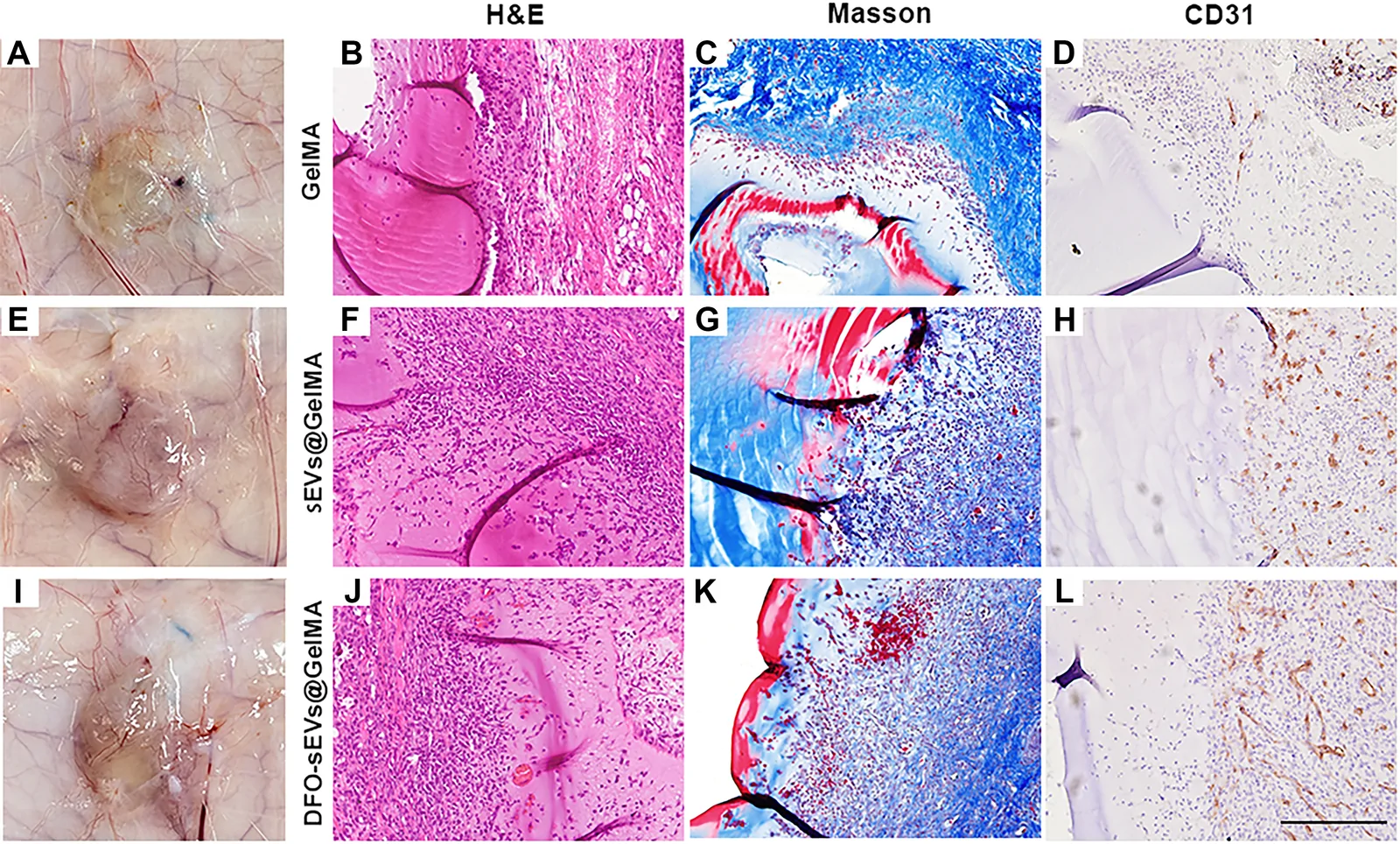
Subcutaneous implantation of DFO-sEVs-loaded GelMA hydrogel in rats. (A, E, and I) Gross views of the implants one week after implantation; (B, F, and J) Histological evaluation (H&E staining); (C, G, and K) Masson’s trichrome; (D, H, and L) CD31 immunohistochemistry. S represents the location of the GelMA hydrogel scaffold; the black arrows indicate the newly formed vessels, and red stars indicate the CD31-positive sites. Each scaffold group contained four independent implantation sites (*n* = 4). Representative histological images are shown for each scaffold group; Scale bar = 200 μm. CD31: Cluster of differentiation 31; DFO: deferoxamine; GelMA: gelatin methacryloyl; H&E: hematoxylin and eosin; sEVs: small extracellular vesicles.

## DISCUSSION

Modulating the culture environment (including biochemical and biophysical cues) of stem cells is an effective approach to regulating the functionality of their secretome and derived sEVs^[13]^. For example, three-dimensional MSC spheroid culture increased the secretion of multiple angiogenesis-associated molecules, such as VEGF, Interleukin-6 (IL-6), and placental growth factor, and conditioned medium from these spheroids enhanced endothelial colony-forming cell migration and tube formation compared with conditioned medium from two-dimensional cultures^[16]^. Hypoxia-preconditioned SHED-derived sEVs likewise enhanced angiogenesis by transferring let-7f-5p and miR-210-3p^[17]^. In contrast to modulating the culture environment, direct drug treatment offers a more stable means of regulating secretome function^[13,18]^. For example, EVs stimulated with a nitric oxide-releasing polymer were enriched in VEGF and miR-126, exhibited enhanced pro-angiogenic activity, and improved neovascularization and limb function in a murine hindlimb ischemia model^[19]^. Herein, we investigated the effects of DFO, a classic hypoxia mimetic, on the secretome and sEVs of SHED.

The conditioned medium experiments in this study were used as an initial screening step to evaluate the overall paracrine effects of DFO-pretreated SHED. Conditioned medium is composed of diverse bioactive components, including soluble proteins, cytokines, growth factors, metabolites, and extracellular vesicles^[2,8]^; therefore, its biological effects cannot be solely attributed to sEVs without additional evidence, such as sEV depletion, fractionation, or rescue experiments. Accordingly, we avoided over-attributing CM-mediated effects to sEVs. sEVs were selected for subsequent investigation because they represent an important bioactive fraction of the MSC secretome and have been shown to mediate MSC paracrine effects in injury repair models^[13,20]^. In addition, sEVs can carry functional proteins and RNAs within membrane-enclosed vesicles^[20-22]^, supporting their role as defined cell-free bioactive components.

Adequate vascularization contributes to tissue repair and regeneration by supporting local perfusion, oxygen delivery, nutrient supply, and tissue integration. Previous studies have shown that pro-angiogenic intervention can accelerate diabetic wound healing, whereas impaired endothelial angiogenic responses are associated with delayed wound repair^[23-25]^. Similar to a hypoxic environment, DFO can upregulate HIF-1α and its downstream key angiogenic factors. This study demonstrates that 5 μM DFO did not impair SHED metabolic activity and enhanced SHED migration, while the secretome derived from DFO-pretreated SHED significantly promoted HUVECs migration and tube formation. RNA-seq analysis further suggests that DFO may influence multiple pathways related to hypoxia response, cell adhesion, and inflammatory signaling in SHED, thereby conferring superior functional properties to their sEVs. These findings suggest that DFO may modulate the cargo composition of sEVs, such as specific miRNAs or proteins, providing a potential strategy to enhance sEV functionality.

Dysregulated inflammation is a critical factor that impedes tissue regeneration^[26,27]^. During tissue repair and regeneration, conditions such as diabetes can lead to chronic inflammation and eventual healing failure^[26,28,29]^. In this study, DFO-sEVs induced the polarization of M1 macrophages toward an M2-like reparative state. Transitioning from the necessary early inflammatory phase to the repair stage in a timely manner is crucial, and M2 macrophages contribute significantly to anti-inflammatory responses and matrix remodeling^[29]^. Thus, DFO-sEVs may synergistically promote tissue regeneration through a dual mechanism of “promoting angiogenesis” and “modulating the immune microenvironment”.

Although this study provides preliminary evidence for the dual functions of pro-angiogenesis and immunomodulation of DFO-sEVs, some limitations remain. First, our *in vivo* evaluation was limited to an ectopic subcutaneous implantation model in normal rats, which provides preliminary evidence for vascularization and tissue integration but does not fully demonstrate therapeutic efficacy in an orthotopic tissue defect or disease model. Further studies using diabetic wound models or tissue defect models with impaired angiogenesis are required. Second, in the current work, we observed notable functional differences between DFO-sEVs and previously reported hypoxia-preconditioned sEVs^[10]^, particularly in the activation of ferroptosis-related pathways, the regulatory mechanisms of which warrant further investigation. Overall, this study suggests that DFO preconditioning can enhance the pro-angiogenic and immunomodulatory potential of SHED-derived sEVs, providing preliminary evidence for developing functionalized sEV-based strategies for vascularized tissue repair.

### Conclusion

In the current work, we found that DFO preconditioning enhanced the pro-angiogenic and immunomodulatory properties of sEVs derived from SHED. In particular, 5 μM DFO-sEVs promoted endothelial cell migration and tube formation, modulated macrophages toward a pro-healing phenotype, and supported vascularization and tissue integration in an ectopic subcutaneous implantation model. These findings support DFO preconditioning as a potential strategy for enhancing the functional properties of sEVs for vascularized tissue repair.

## DECLARATIONS

### Availability of data and materials

The original contributions presented in the study are included in the article/Supplementary Materials; further inquiries can be directed to the corresponding authors.

### AI and AI-assisted tools statement

During the preparation of this manuscript, the AI tool ChatGPT (version GPT-5.4 Thinking, released 2026-03-05) was used solely for language editing. The tool did not influence the study design, data collection, analysis, interpretation, or the scientific content of the work. All authors take full responsibility for the accuracy, integrity, and final content of the manuscript.

### Copyright

© The Author(s) 2026.
