# Supplementary material for: Small extracellular vesicles from deferoxamine-primed stem cells promote angiogenesis and tissue integration
Source: Extracell Vesicles Circ Nucl Acids. 2026 Sep 8;7(3):1428–44. doi: 10.20517/evcna.2026.48 (PMC13673346; doi:10.20517/evcna.2026.48)
Supplement: Supplementary file 1 [file evcna-7-3-1428-SupplementaryMaterials.pdf]

Supplementary Materials

Small extracellular vesicles from deferoxamine-primed stem cells promote angiogenesis and tissue integration

Jingyi Sang[1], Yuqing Dong[1], Xiaoyue Zhang[2], Xiaojing Yuan[1], Yike Gao[1], Rui Song[1], Xiya Liu[1], Linxue Zhang[1], Zuoying Yuan[3], Yuming Zhao[1]

[1]Department of Pediatric Dentistry, Peking University School and Hospital of Stomatology, National Center for Stomatology, National Clinical Research Center for Oral Diseases, National Engineering Research Center of Oral Biomaterials and Digital Medical Devices, Beijing Key Laboratory of Digital Stomatology, Research Center of Engineering and Technology for Computerized Dentistry, Ministry of Health, NMPA Key Laboratory for Dental Materials, Beijing 100081, China.
[2]College of Fisheries, Guangdong Ocean University, Zhanjiang 524088, Guangdong, China.
[3]Center of Basic Medical Research, Institute of Medical Innovation and Research, Peking University Third Hospital, Beijing 100191, China.

Correspondence to: Dr. Xiaojing Yuan, Prof. Yuming Zhao, Department of Pediatric Dentistry, Peking University School and Hospital of Stomatology, National Center for Stomatology, National Clinical Research Center for Oral Diseases, National Engineering Research Center of Oral Biomaterials and Digital Medical Devices, Beijing Key Laboratory of Digital Stomatology, Research Center of Engineering and Technology for Computerized Dentistry, Ministry of Health, NMPA Key Laboratory for Dental Materials, Beijing 100081, China. E-mail: xjy@bjmu.edu.cn; zhaoyuming@pkuss.bjmu.edu.cn; Prof. Zuoying Yuan, Center of Basic Medical Research, Institute of Medical Innovation and Research, Peking University Third Hospital, Beijing 100191, China. E-mail: zy_yuan@pku.edu.cn

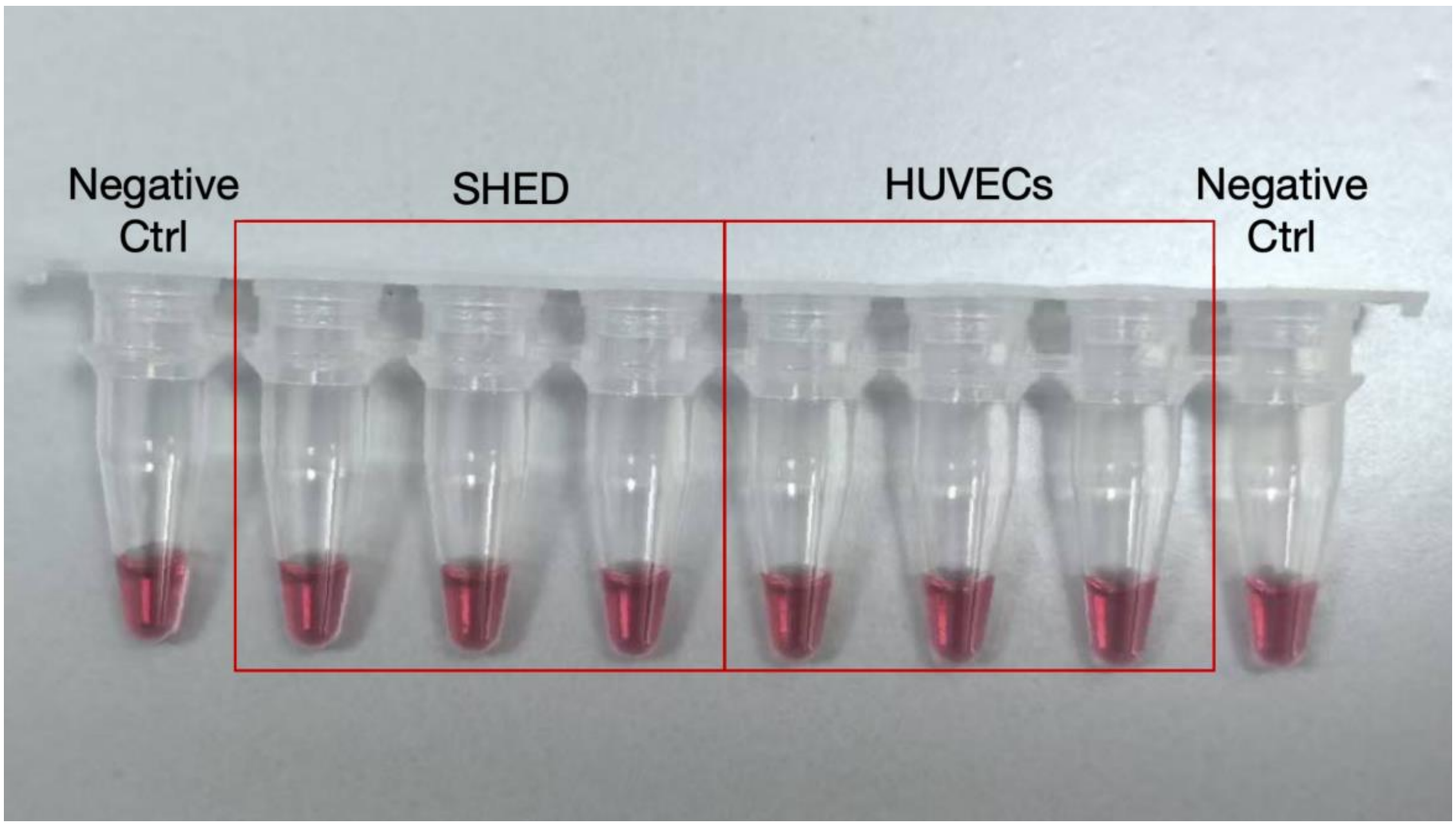

Supplementary Figure 1. Mycoplasma detection for SHED and HUVECs.

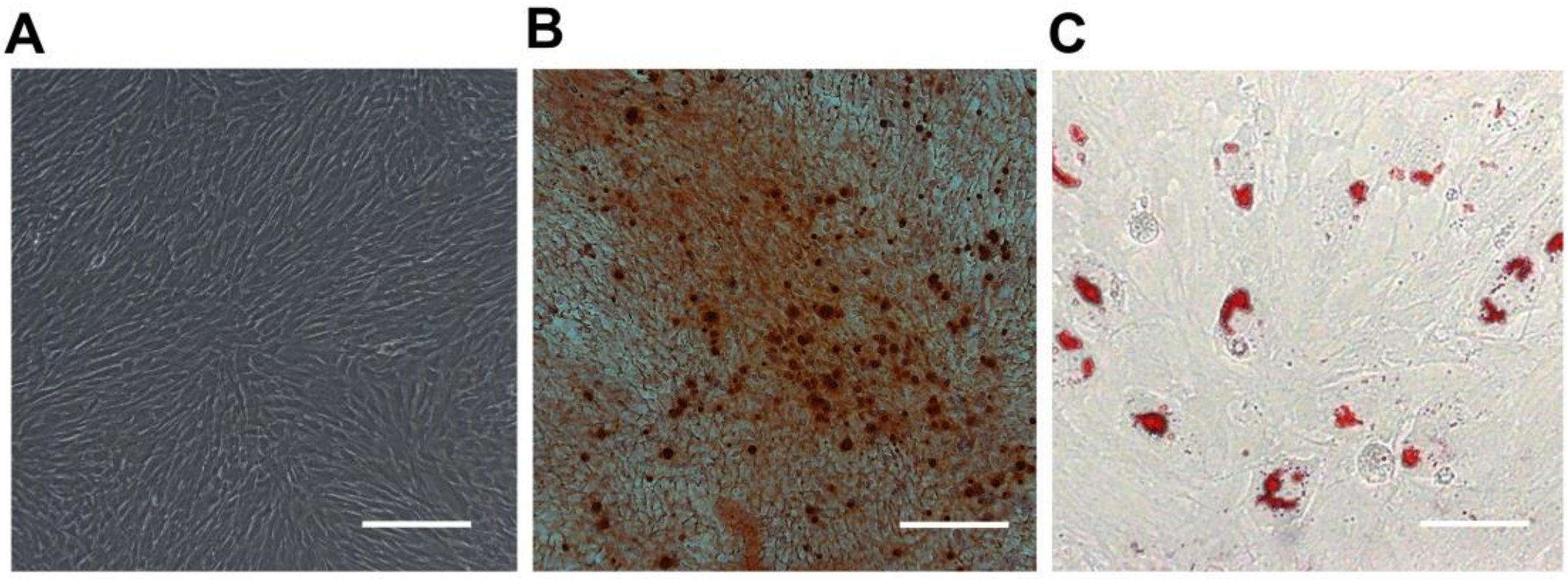

D

	CD45	CD73	CD29
Mean +/-SD	0.4167+/-0.1474%	97.59+/-2.913%	99.65+/-0.1582%

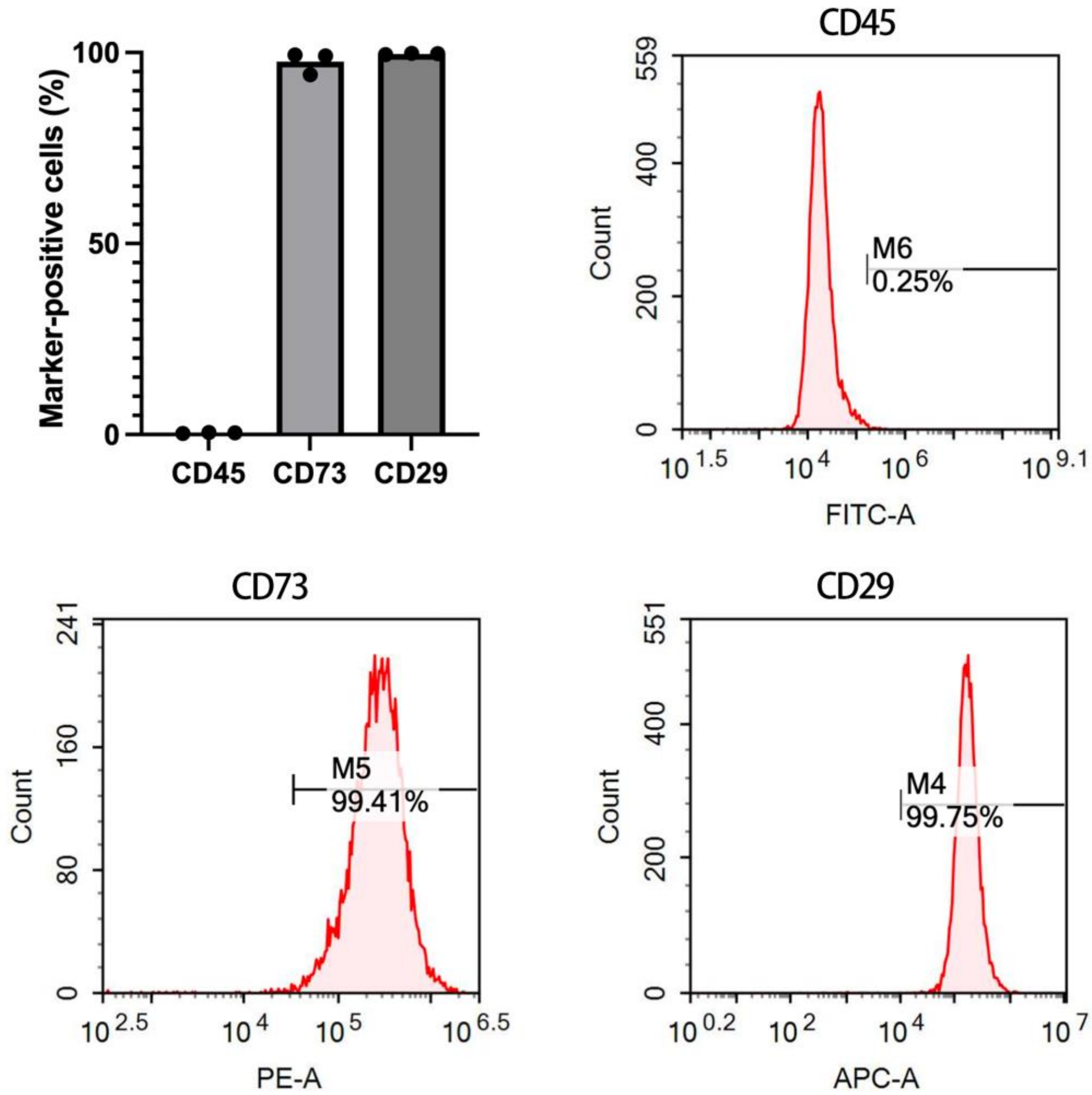

Supplementary Figure 2. Identification and characterization of SHED. Representative images and flow cytometry profiles are shown. (A) Morphological observation of SHED (Scale bar: 200 μm); (B and C) Osteogenic and adipogenic differentiation of SHED assessed by Alizarin Red S (Scale bar: 200 μm) and Oil Red O staining, respectively (Scale bar: 50 μm); (D) Flow cytometric analysis of SHED surface markers CD45, CD73, and CD29. Quantitative data are presented as mean ± SD (*n* = 3 biological replicates).

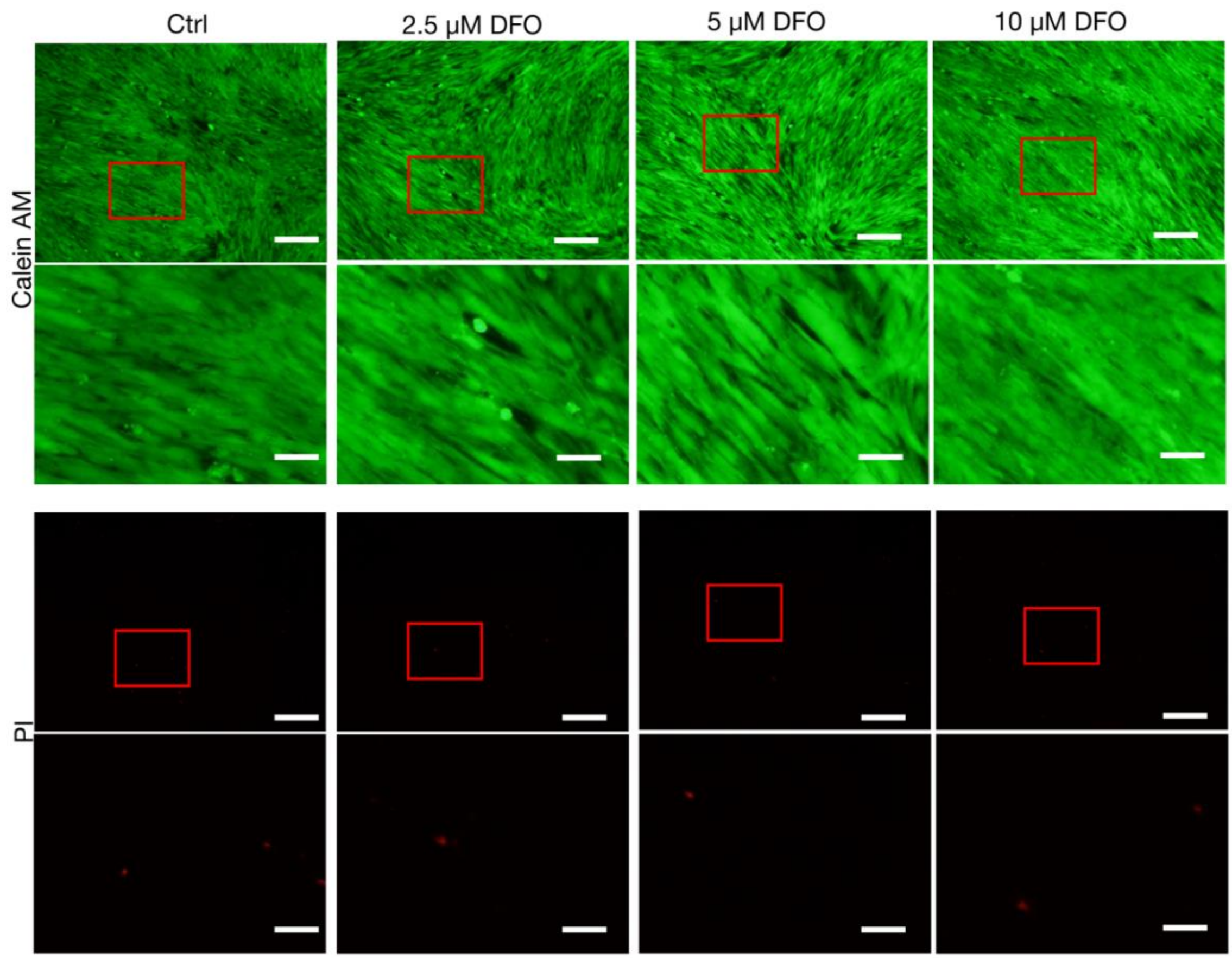

Supplementary Figure 3. Effects of DFO preconditioning on SHED viability. Representative Calcein AM/PI staining images of SHED treated with different concentrations of DFO. Live cells were stained green by Calcein AM, whereas dead cells were stained red by PI. The enlarged images correspond to the red boxed regions in the upper panels (*n* = 3 independent biological replicates, Scale bar: 200 μm for the upper panels and 50 μm for the enlarged panels).

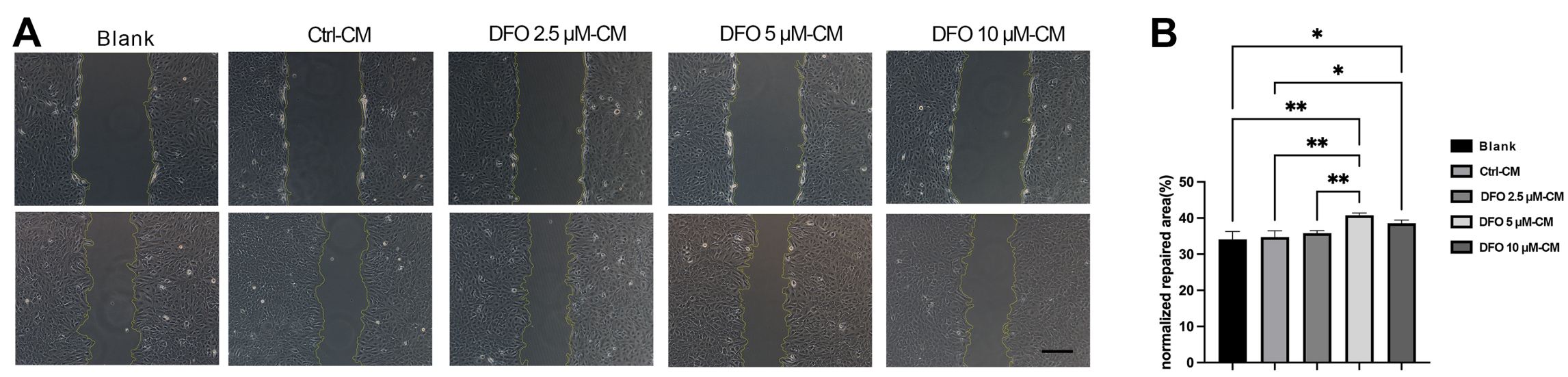

Supplementary Figure 4. Effects of DFO Preconditioning on the Secretome of SHED. (A) HUVECs migration assessed by wound healing assay (Scale bar: 200 μm), (B) with corresponding quantitative analysis. Data are presented as mean ± SD from at least three independent biological replicates. Statistical analysis was performed using one-way ANOVA followed by Tukey's HSD post-hoc test. *$P < 0.05$, **$P < 0.01$, ***$P < 0.001$, ****$P < 0.0001$.

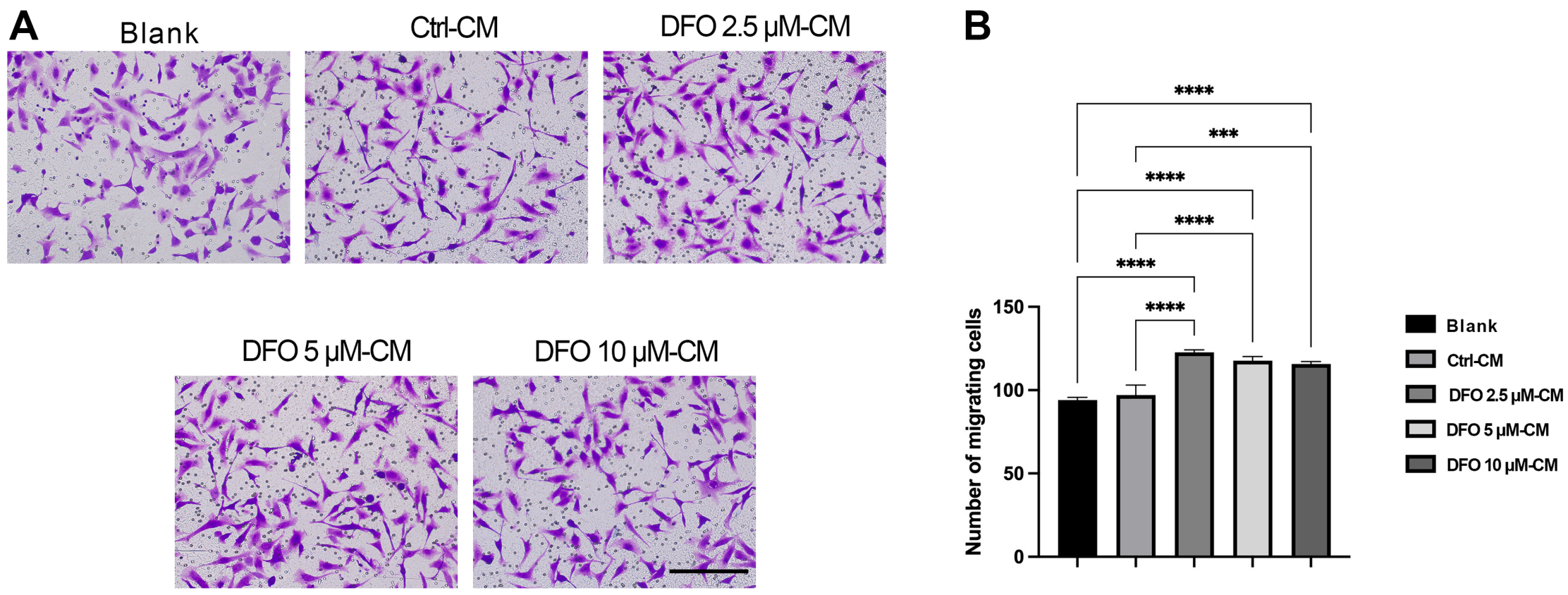

Supplementary Figure 5. Effects of DFO Preconditioning on the Secretome of SHED. (A) HUVECs migration assessed by transwell assay (Scale bar: 200 μm), (B) with corresponding quantitative analysis. Data are presented as mean ± SD from at least three independent biological replicates. Statistical analysis was performed using one-way ANOVA followed by Tukey's HSD post-hoc test. *$P < 0.05$, **$P < 0.01$, ***$P < 0.001$, ****$P < 0.0001$.

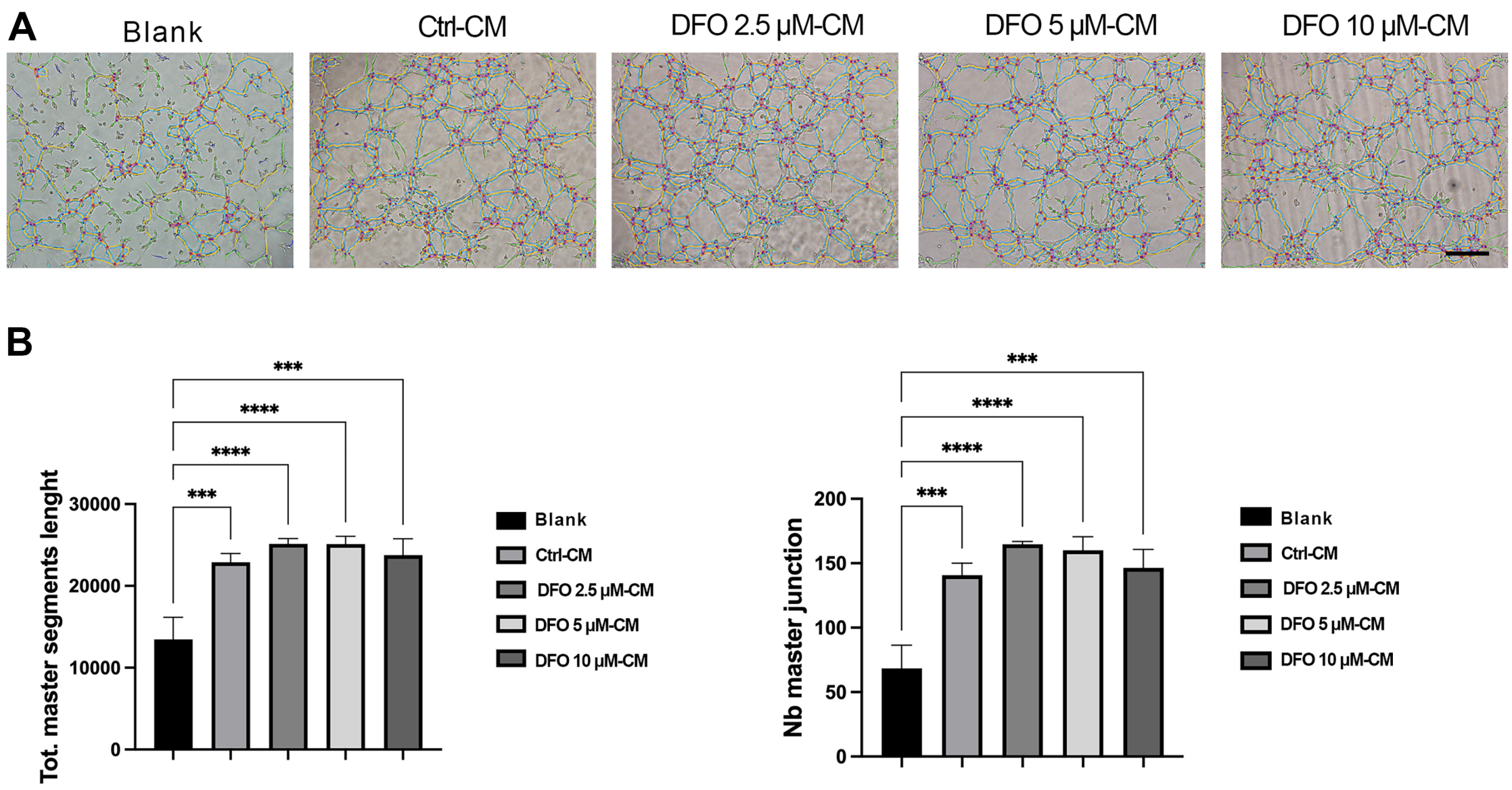

Supplementary Figure 6. Effects of DFO Preconditioning on the Secretome of SHED. (A) Matrigel tube formation assay of HUVECs (Scale bar: 200 μm) and (B) corresponding quantitative analysis of total master segments length and number of master junctions. Data are presented as mean ± SD from at least three independent biological replicates. Statistical analysis was performed using one-way ANOVA followed by Tukey's HSD post-hoc test. *$P < 0.05$, **$P < 0.01$, ***$P < 0.001$, ****$P < 0.0001$.
